# The Immediate Pulmonary Disease Pattern following Exposure to High Concentrations of Chlorine Gas

**DOI:** 10.1155/2013/325869

**Published:** 2013-12-09

**Authors:** Pallavi P. Balte, Kathleen A. Clark, Lawrence C. Mohr, Wilfried J. Karmaus, David Van Sickle, Erik R. Svendsen

**Affiliations:** ^1^Arnold School of Public Health, University of South Carolina, 800 Sumter Street, Room 210, Columbia, SC 29208, USA; ^2^Medical University of South Carolina, 135 Cannon Street, Suite 405, P.O. Box 250838, Charleston, SC 29425, USA; ^3^Division of Epidemiology, Biostatistics, and Environmental Health, School of Public Health, University of Memphis, 301 Robison Hall, 3825 De Soto Avenue, Memphis, TN 38152, USA; ^4^Asthmapolis, 612 W. Main Street, Suite 201, Madison, WI 53703, USA; ^5^Department of Global Environmental Health Sciences, Tulane University School of Public Health and Tropical Medicine, 1440 Canal Street, Suite 2100, New Orleans, LA 70112, USA

## Abstract

*Background*. Classification of pulmonary disease into obstructive, restrictive, and mixed patterns is based on 2005 ATS/ERS guidelines and modified GOLD criteria by Mannino et al. (2003), but these guidelines are of limited use for simple spirometry in situations involving mass casualties. *Aim*. The purpose of this study was to apply these guidelines to patients who underwent simple spirometry following high concentration of chlorine gas inhalation after a train derailment in Graniteville, South Carolina. *Methods*. We retrospectively investigated lung functions in ten patients. In order to classify pulmonary disease pattern, we used 2005 ATS/ERS guidelines and modified GOLD criteria along with our own criteria developed using available simple spirometry data. *Results*. We found predominant restrictive pattern in our patients with both modified GOLD and our criteria, which is in contrast to other chlorine exposure studies where obstructive pattern was more common. When compared to modified GOLD and our criteria, 2005 ATS/ERS guidelines underestimated the frequency of restrictive disease. *Conclusion*. Diagnosis of pulmonary disease patterns is of importance after irritant gas inhalation. Acceptable criteria need to be developed to evaluate pulmonary disease through simple spirometry in events leading to mass casualty and patient surge in hospitals.

## 1. Introduction

Chlorine gas is one of the most commonly used industrial chemicals and is a potential weapon of mass destruction [1–8]. The health effects of chlorine inhalation depend on chlorine concentration and duration of exposure. If inhaled in low concentration (<50 ppm) chlorine gas is known to cause mild irritation of mucus membranes, coughing, choking, and shortness of breath [9, 10]. Exposure to high concentrations (>50 ppm) may damage the lower respiratory tract and lung parenchyma causing complications such as rapid development of interstitial pneumonia, pulmonary edema, and death due to progressive respiratory failure [9–11]. Several studies have shown decrease in lung function after acute inhalation of chlorine gas, but very few studies attempted to determine pulmonary disease pattern in these patients [11–14]. Although obstructive pulmonary disease was most commonly observed in all these studies, restrictive and mixed pulmonary disease were also seen in a few studies [12–14].

At present, 2005 ATS/ERS task force guidelines based on NHANES III data are considered the “gold standard” and are used universally to provide guidance to physicians and hospital based pulmonary function tests (PFTs) laboratories for interpreting PFTs [15]. These guidelines are based on availability of plethysmography to determine total lung capacity (TLC). On the other hand, Mannino et al. used a modification of the Global Initiative for Chronic Obstructive Lung Disease (GOLD) criteria to determine pulmonary disease patterns in NHANES I cohort based on spirometry data alone [16].

The modified GOLD criteria [16] are a very useful tool for diagnosing and assessing the severity of COPD through simple spirometry without lung volume measurements in clinical settings [17]. However, there are no specific guidelines for diagnosing mixed pattern. The 2005 ATS/ERS guidelines [15] cannot be used with spirometry data alone due to lack of information on lung volumes.

Both ATS/ERS guidelines and modified GOLD criteria are extremely valuable to diagnose, characterize, and assess pulmonary disease in patients routinely admitted to hospitals [17–19]. These guidelines are also useful in diagnosing pulmonary disease in irritant gas exposure events with small number of people affected [20]. But in mass casualty events providing diagnostic PFTs is not possible within days of occurrence [21, 22]. In emergency situations like these where a large number of people are affected, healthcare services are more concentrated on making quick medical evaluation and providing appropriate emergent treatment. There are rarely major resources available to provide diagnostic testing like complete PFTs in such situations. Therefore, assessment of severity of pulmonary disease has to be based on available resources like spirometry. However, at present there are no known universally acceptable criteria or guidelines available that can be used to diagnose pulmonary disease during mass casualty or in situations where healthcare resources are limited due to patient surge in emergency departments. Irritant gas exposure is known to have long term sequelae, so it is also important to know different pulmonary disease patterns and the pathophysiology behind it for long term follow-up [23, 24].

The purpose of this study was to apply 2005 ATS/ERS and Mannino et al. (2003) guidelines along with our own criteria to determine pulmonary function patterns in ten patients who underwent simple spirometry following high concentration of chlorine gas inhalation after a train derailment in Graniteville, South Carolina.

## 2. Methods

### 2.1. Graniteville Train Accident

On January 6, 2005, a train derailment and tanker breach led to the largest chlorine spill in the United States history, releasing approximately 60 tons of chlorine gas in Graniteville, South Carolina. A report was published earlier describing exposure to the community, evacuation, and emergency medical response [21]. There were nine immediate fatalities and 525 emergency room visits with 71 hospitalizations initially reported. Twenty-three of these patients were intubated and placed on mechanical ventilation. The remaining 48 were admitted to acute care with various clinical manifestations attributed to high concentration of chlorine gas inhalation. All patients were admitted within the first 24 hours after exposure. The initial evaluation included physical examination, chest radiography, and arterial blood gas determinations. Spirometry was performed on ten patients. A detailed description of sociodemographic characteristics, clinical signs and symptoms, hematological and biochemical findings, radiographic findings, and treatment in all 71 patients admitted to hospital was previously published [22].

### 2.2. Study Population and Protocol

We conducted a case series study of the ten patients who underwent simple spirometry testing while being admitted in the hospital. The data used for this report were part of a larger public health effort directed by South Carolina Department of Health and Environmental Control (SCDHEC) which focused on recovery and mitigation of disease in the exposed Graniteville community. A retrospective chart review was conducted under the public health authority within declared emergency situations. We accessed a de-identified SCDHEC database from that public health chart review. This study was approved by both SCDHEC and University of South Carolina (USC) institutional review boards (IRB).

### 2.3. Criteria for Classification of Pulmonary Disease Pattern

All spirometry tests were performed within one week of admission (on an average 5th day after admission). Spirometry tests were performed according to ATS/ERS guidelines [15]. The predicted and lower limits of normal (LLN) values were calculated using NHANES III prediction equations [25]. In order to classify pulmonary disease patterns in these patients we applied two widely used criteria: 2005 ATS/ERS task force guidelines [15] which are derived from NHANES III data and require lung volume parameters and criteria used by Mannino et al. which are based on simple spirometry [16]. In our study we had simple spirometry data without any lung volume parameters on ten patients. As 2005 ATS/ERS guidelines require lung volume parameters to diagnose pulmonary disease pattern and Mannino et al. (2003) did not have any specified criteria to diagnose mixed pattern, we were unable to use these two criteria to their full extent in our study. We therefore developed criteria that facilitate diagnosis of all three pulmonary disease patterns using available spirometry data. We do not consider these criteria as guidelines to be used in mass casualty events, as these are based on data from only ten patients. We use these criteria here as only a tool to get a complete picture of pulmonary disease pattern in the patients admitted to hospital and to compare them with existing guidelines (Table 1).

### 2.4. Statistical Analyses

This is a descriptive epidemiological study with a small sample size. We calculated the agreement between different criteria used to classify pulmonary disease patterns. The data analysis for this paper was generated using SAS 9.2. (SAS Institute Inc., Cary, NC, USA).

## 3. Results

Of the ten patients we evaluated, five were admitted to the intensive care unit (ICU), and two of these required intubation and mechanical ventilation due to acute lung injury. There were nine males and one female with a mean age of 44.7 ± 16.1 years. Among the ten patients four were current smokers, five were nonsmokers, and one was a former smoker (Table 2). Presenting symptoms specific to chlorine exposure were shortness of breath, coughing, choking, chest pain, productive cough, eye irritation, nose irritation and sore throat (Table 3). Symptoms less specific to chlorine exposure were dizziness, headache, nausea, and vomiting. On physical examination tachypnea and tachycardia were the most common findings. Other notable findings on auscultation were wheeze, crackles, and decreased breath sounds.

Mean oxygen saturation upon admission to emergency department was 81.1 ± 18.1% (Table 4). Mean white blood cell (WBC) count in all patients was 13.91 ± 3.25 × 10^3^ cells/*μ*L within the first 24 hours with an elevated neutrophil count in six patients, mean 79.7 ± 4.8%. The average blood pH and PCO_2_ in nine patients were 7.32 ± 0.08 and 47.42 ± 9.02 mm of Hg, respectively. Chest radiography was performed on all patients. Seven patients showed evidence of pulmonary edema and one showed increased bronchovascular markings, while two patients had a normal chest radiograph. Electrocardiography was performed on six patients and three showed nonspecific T-wave changes.

All patients received warm humidified oxygen on arrival at the emergency department along with inhaled short acting beta receptor agonists. Additionally, all patients were treated with either inhaled or oral steroids, six were treated with inhaled ipratropium bromide, and seven were treated with antibiotics. One patient also received intravenous sodium bicarbonate due to severe acid-base imbalance. The average stay in the hospital was 8.1 ± 5.4 days.

All spirometry tests were done within the first week (on an average 5th day after admission) of hospitalization after the patient was stabilized. Mean FVC and FEV_1_ were 2.51 ± 0.39 L and 1.84 ± 0.36 L, respectively (Table 4). Mean FEV_1_ percent predicted was 53.4 ± 13.4%, mean FVC percent predicted was 57.2 ± 11.6%, and mean FEV_1_/FVC ratio was 0.76 ± 0.09. Using 2005 ATS/ERS task force guidelines [13], we found an obstructive pattern in three and restrictive pattern in two patients. We were not able to determine the primary pulmonary disease pattern in the remaining five patients as information on TLC was not available (Table 5). Based on guidelines used by Mannino et al. (2003), we found a restrictive pattern in eight patients, and obstructive pattern in two patients. After applying our criteria based, we found a restrictive pattern in seven patients, obstructive pattern in two patients and mixed pattern in one patient (Table 5). The agreement between our criteria and guidelines provided by Mannino et al. (2003) was 75%. Agreement between our criteria and 2005 ATS/ERS task force guidelines resulted in only 16.7% agreement which is expected as we did not have lung volume parameters to use 2005 ATS/ERS guidelines to full extent. Among current smokers, three had restrictive and one had obstructive pattern, while among nonsmokers three had restrictive, one had mixed, and one had an obstructive pattern. One former smoker showed a restrictive pattern.

## 4. Discussion

Chlorine is one of the most commonly produced chemicals in the United States and the most common cause of household exposure to irritant gases resulting in lung injury [9]. Chlorine is also considered as a hazardous material with a constant threat of industrial accidental exposure and as a terrorist weapon [26]. Medical management decisions for acute chlorine inhalation are subjective and largely depend on presenting signs and symptoms and oxygen saturation [27]. Acute injury from chlorine gas exposure may induce chronic inflammation and fibrosis, which may then influence the rate of functional decline [24, 28] and lead to restrictive lung disease [29]. It is important to determine pulmonary disease pattern after acute inhalation of chlorine gas because the treatment modalities differ in obstructive and restrictive lung disease. Obstructive disease is primarily treated with bronchodilators and inhaled corticosteroids depending on the stage of disease whereas treatment of restrictive disease mainly focuses on immunosuppression using oral corticosteroids in early stages and drugs like cyclophosphamide in late stages [29].

In the present study, all ten patients exhibited symptoms consistent with high concentration of irritant gas exposure and moderate to severe hypoxemia, tachypnea, and tachycardia. Eight patients demonstrated adventitious breath sounds associated with adverse clinical symptoms such as dyspnea, wheezing, coughing, and chest pain. Pulmonary edema and increased bronchovascular markings were common on chest radiographs. After applying our criteria to determine pulmonary disease patterns we found that restrictive pattern (*n* = 7) was more common than the obstructive (*n* = 2) and mixed (*n* = 1) pattern. All patients received humidified oxygen and short acting beta agonists. Treatment should have improved pulmonary function after one-week period. However, we found that all patients who underwent pulmonary function testing had abnormal lung function. As mentioned earlier, obstructive lung disease is more common after exposure to high concentration of chlorine gas [12–14]. In contradiction to other studies we found that restrictive pulmonary disease was more common than obstructive pulmonary disease. We found no past medical history for preexisting underlying lung disease during chart review. Additionally, there were no specific differences in pulmonary disease patterns among smokers and nonsmokers.

There are several limitations to our study. Firstly, our sample size was very small, too small to draw any definite conclusions. Secondly, since the data came from a retrospective review of medical charts for public health reporting purposes, the information retrieved was very limited. Thirdly, TLC measurements were not available. Finally, we also did not have baseline pulmonary function measurements for comparison.

Several studies have examined the acute effects of high concentration of chlorine inhalation on pulmonary functions with differing results. Charan et al. studied 19 people exposed to chlorine gas in an accident in a pulp mill [14]. They found an obstructive disease pattern in ten out of 19 patients admitted within the first 24 hours. After a period of 10 days, only seven out of ten patients showed airflow obstruction. In another study Moulick et al. studied 82 patients exposed to chlorine leaked from a storage tank at a factory in Mumbai, India [12]. The investigators found an obstructive pattern in 22 (26.8%), restrictive patter in three (3.7%), and mixed pattern in 44 (53.4%) cases within the first 48 hours. Similarly, Barret and Faure after studying 129 patients admitted in Grenoble, France, who underwent spirometry in the acute period after exposure to chlorine found an obstructive pattern in 63%, restrictive pattern in 30%, and mixed pattern in 7% [13]. However, we were unable to obtain information on specific guidelines used for classifying pulmonary disease pattern in the above-mentioned studies. So, we applied three different criteria to classify pulmonary disease patterns using simple spirometry: (1) 2005 ATS/ERS guidelines; (2) modified GOLD criteria used by Mannino et al. (2003); and (3) Graniteville criteria. We found predominant restrictive pattern in our patients by using both modified GOLD and Graniteville criteria, in contrast with above-mentioned studies. The obstructive pattern after chlorine inhalation can be attributed to the irritating action of hypochlorous and hydrochloric acid along with bronchospasm [30, 31]. The restrictive patterns seen in our review could be due to neutrophil sequestration in the respiratory system (as evidenced by elevated neutrophil counts) leading to acute, early onset of inflammation and pulmonary edema [30]. Accumulation of fluid in the alveoli would produce restrictive pattern [30]. In this study, we found radiographic evidence of pulmonary edema in six out of seven patients with a restrictive spirometry pattern and four of them had elevated neutrophil counts. While comparing our criteria to Mannino et al. (2003), we saw strong agreement on obstructive and restrictive disease patterns but no agreement on mixed pattern. With 2005 ATS/ERS guidelines, we agreed on two restrictive patterns and one obstructive pattern mainly due to unavailability of TLC measurements and also due to small sample size. However, our findings are consistent with Aggarwal and Agarwal [32], who studied simple spirometry (without any lung volume measurements) in 2,527 patients prospectively over a period of six months at the Postgraduate Institute of Medical Education and Research, Chandigarh, India. They compared 2005 ATS/ERS task force guidelines with 1991 ATS guidelines and reported that the 2005 ATS/ERS guidelines underestimated the frequency and severity of the restrictive disease pattern [32].

Any mass casualty disaster can overtax and paralyze the local health care system as it demands emergency medical responses on a large scale. Irritant or chemical gas exposure events are known to occur all around the world. The most notable examples are Bhopal Gas Tragedy [33, 34] and Chernobyl accident [35]. Not all healthcare institutions have infrastructure to routinely perform PFTs and if they do only few have facilities to perform a complete diagnostic work-up with lung volume measurements. Most of the energy focuses on providing symptomatic relief to patients with minor symptoms and stabilization of more critical patients, thus limiting resources for providing diagnostic testing [36–38]. However, after the initial spirometry patients should be followed up with full diagnostic PFTs. Several studies have suggested that the exposure to high concentration of irritant gas exposure may lead to long term pulmonary complications [14, 23, 39]. We are aware of the hesitancy to perform diagnostic testing immediately after an event. But serial complete PFTs can alert the physician as to the inflammatory processes that occur along with remodeling after the obstructive pattern subsides [23, 24]. Additionally, acceptable criteria should be developed to diagnose restrictive pattern through simple spirometry to aid in therapeutic recommendations.

## 5. Conclusions

We found reduction in lung function and predominant restrictive pulmonary pattern in our patients on simple spirometry done within the first week of admission to hospital after exposure to high concentrations of chlorine gas. This finding was in contrast to other studies done in patients exposed to chlorine gas where obstructive disease was more prominent. There is a need to develop acceptable criteria to evaluate pulmonary disease using parameters available through simple spirometry in events such as irritant gas exposure leading to mass casualty and patient surge in hospitals.

## Figures and Tables

**Table 1:** Comparison of different criteria for classification of pulmonary disease patterns.

Pulmonary disease pattern	2003 Mannino et al. guidelines [16]	2005 ATS/ERS task force guidelines^#^ [15]	Graniteville criteria
Obstructive	Mild: FEV_1_/FVC ratio < 70%, FEV_1_ ≥ 80% of predicted Moderate: FEV_1_/FVC ratio < 70%, FEV_1_ ≥ 50% but < 80% of predicted Severe: FEV_1_/FVC ratio < 70%, FEV_1_ < 50% of predicted	FEV_1_/VC ratio below LLN*	FEV_1_/FVC ratio ≤ 0.70 and normal, reduced, or increased FEV_1_ and FVC percent predicted

Restrictive	FEV_1_/FVC ratio ≥ 70 and FVC < 80% of predicted	TLC below LLN or FEV_1_/VC ratio ≥ 85 and a reduced VC	FEV_1_/FVC ratio ≥ 0.75, FEV_1_ and FVC < 75% of predicted

Mixed	No criteria reported	FEV_1_/VC ratio and TLC below LLN	FEV_1_/FVC ratio > 0.70 and < 0.75 and both FEV_1_ and FVC < 75% predicted

^#^Vital capacity (VC) measures were unavailable, so we used FEV_1_/FVC ratio instead of FEV_1_/VC ratio as suggested by 2005 ATS/ERS guidelines.

*Lower limit of normal.

**Table 2:** Sociodemographic characteristics of patients who underwent spirometry.

Characteristic	*N* = 10
Age (years), mean ± S.D.	44.7 ± 16.1
Gender, *n *	
Male	9
Female	1
Race, *n *	
Caucasian	6
African American	4
Smoker, *n *	
Current	4
Former	1
Nonsmoker	5

**Table 3:** Presenting symptoms and physical findings in patients who underwent spirometry.

Variable	*N*
Symptoms:	
Sore throat	35
Cough	9
Nose irritation	8
Shortness of breath	7
Choking	6
Chest pain	6
Productive cough	5
Dizziness	3
Headache	4
Eye irritation	4
Nausea/vomiting	2
Physical findings:	
Tachypnea	10
Tachycardia	9
Crackles	5
Wheeze	6
Decreased breath sounds	2

**Table 4:** Mean and standard deviation of pulmonary function parameters, hematological and biochemical findings, and oxygen saturation.

Variable	*N*	Mean ± S.D.
Pulmonary function tests (done within one week):		
Measured FVC	10	2.51 ± 0.39 mL
Measured FEV_1_	10	1.84 ± 0.36 mL
FVC% predicted	10	57.2 ± 11.6%
FEV_1_% predicted	10	53.4 ± 13.4%
FEV_1_/FVC ratio	10	0.76 ± 0.09
LLN FVC	10	3.72 ± 0.68 mL
LLN FEV_1_	10	2.97 ± 0.57 mL
LLN FEV_1_/FVC ratio	10	0.70 ± 0.04
Hematological and biochemical findings (done within first 24 hours):		
Total WBC count	10	13.9 ± 3.2 × 10^3^ cells/*µ*L
Neutrophil count	6	79.7 ± 4.8%
Blood pH	9	7.32 ± 0.08
Blood CO_2_	9	47.42 ± 9.02 mm Hg
Blood HCO_3_	7	54 ± 79.86 mEq/L
Oxygen saturation (done in emergency department):		
Overall	10	81.1 ± 18.1%
With pulmonary edema	7	77.9 ± 21.2%
Without pulmonary edema	3	88.7 ± 3.8%

**Table 5:** Pulmonary function pattern and radiographic findings in patients who underwent spirometry.

Patient	Smoking status	Percent predicted		FEV_1_/FVC ratio	LLN FEV_1_/FVC ratio	Hematology		Chest radiographs		Pulmonary function pattern		
		FVC	FEV_1_			Total WBC^#^	Neut*%	PE	IVM	ATS/ERS Task force^*£*^	Mannino et al.^¥^ [16]	Graniteville criteria^$^
1	Current	59	43	0.55	0.63	9.3	73.8	Yes	Yes	O	Severe O	O
2	Current	40	37	0.75	0.71	14.2	Miss.	Yes	No	U	R	R
3	Current	50	58	0.79	0.67	15.2	Miss.	Yes	No	U	R	R
4	Current	45	45	0.85	0.74	19.7	Miss.	Yes	No	R	R	R
5	Nonsmoker	56	46	0.73	0.76	16	80	Yes	No	O	R	M
6	Nonsmoker	68	63	0.69	0.68	11	75	No	No	U	Moderate O	O
7	Nonsmoker	71	66	0.81	0.71	12.1	86	No	No	U	R	R
8	Nonsmoker	71	75	0.87	0.70	14.4	79.3	No	Yes	R	R	R
9	Nonsmoker	66	64	0.78	0.71	16.9	Miss.	Yes	No	U	R	R
10	Former	46	37	0.81	0.71	10.3	84	Yes	No	U	R	R

^#^×10^3^ cells/*μ*L, *neutrophils.

PE: pulmonary edema; IVM: increased vascular markings; Miss.: missing.

O: obstructive pattern; R: restrictive pattern; M: mixed pattern; U: undetermined pattern.

^*£*^Obstructive pattern: FEV1/VC ratio below LLN; Restrictive pattern: TLC below LLN or FEV_1_/VC ratio ≥ 85% and a reduced VC; mixed: FEV_1_/VC ratio and TLC below LLN.

^¥^Obstructive pattern: mild: FEV_1_/FVC ratio < 70%, FEV_1_ ≥ 80% of predicted, moderate: FEV_1_/FVC ratio < 70%, FEV_1_ ≥ 50%–< 80% of predicted, severe: FEV_1_/FVC ratio < 70%, FEV_1_ < 50% of predicted; restrictive pattern: FEV_1_/FVC ratio ≥ 70% and FVC < 80% of predicted.

^$^Obstructive pattern: FEV_1_/FVC ratio ≤ 0.70 and normal, reduced, or increased FEV_1_ and FVC percent predicted; restrictive pattern FEV_1_/FVC ratio ≥ 0.75, FEV_1_ and FVC < 75% of predicted; mixed: FEV_1_/FVC ratio > 0.70 and < 0.75 FEV_1_ and FVC < 75% predicted.

## Abbreviations

ATS/ERS: American Thoracic Society/European Respiratory Society
GOLD: Global Initiative for Chronic Obstructive Lung Disease
ppm: Parts per million
NHANES: National Health and Nutrition Examination Survey
PFT: Pulmonary function testing
TLC: Total lung capacity
SCDHEC: South Carolina Department of Health and Environmental Control
USC: University of South Carolina
IRB: Institutional review board
ICU: Intensive care unit
LLN: Lower limit of normal
FEV_1_: Forced expiratory volume in one second
FVC: Forced vital capacity
FEV_1_/FVC: Forced expiratory volume in one second/forced vital capacity
VC: Vital capacity
SAS: Statistical Analysis Software
WBC: White blood count.
